# Socio-demographic, behavioral, and biological risk factors of hypertension in Kazakhstan: results of a national study

**DOI:** 10.1186/s12963-026-00454-9

**Published:** 2026-01-14

**Authors:** Yevgeniy Zhukov, Kuanysh Nikatov, Ermek Dyussembekov, Rauan Kastey, Niyazbek Yerniyazov, Mukhtar Korabayev, Darina Menlayakova, Talgat Muminov, Shynar Tanabayeva, Ildar Fakhradiyev, Marat Shoranov

**Affiliations:** 1 S.D.Asfendiyarov Kazakh National Medical University, 94, Tole-bi str., Almaty, 050020 Republic of Kazakhstan; 2 City clinical Hospital No7, Almaty, Kazakhstan; 3 Multidisciplinary hospital named after prof. Kh.Zh.Makazhanov, Karagandy, Kazakhstan; 4 Municipal State Enterprise on the Right of Economic Management “East Kazakhstan region Specialized Medical Center” of Health Care Administration of East Kazakhstan region, Kazakhstan region, Ust-Kamenogorsk, Kazakhstan; 5 College of Medicine, Korea University, Seoul, South Korea

**Keywords:** Hypertension, Risk factors, Modifiable, Non-modifiable, Kazakhstan

## Abstract

**Background:**

Arterial hypertension (AH) is a major contributor to cardiovascular morbidity and mortality worldwide. This study aimed to identify sociodemographic and biological factors associated with hypertension in a nationally representative adult sample in Kazakhstan.

**Methods:**

A cross-sectional WHO STEPS survey (October 2021–May 2022) included 6,720 adults aged 18–69 years from all regions of Kazakhstan. Sociodemographic, behavioral, physical and biochemical data were collected, hypertension was defined by ESC/ESH criteria.

**Results:**

Crude AH prevalence was 16.0% (95% CI 15.1–16.8) and increased sharply with age from 3.0% at 18–24 years to 46.7% at ≥ 65 years (*p* < 0.001). Men had higher systolic (SBP) and diastolic blood pressure (DBP) than women (126/82 vs. 119/79 mmHg, *p* < 0.001) and a less favorable BP profile. Urban residents were younger and more likely to report smoking and alcohol use than rural residents (21.4% vs. 14.8% and 6.5% vs. 3.2%, *p* < 0.001), whereas rural participants had higher BMI, SBP, DBP, total cholesterol and HbA1c (*p* < 0.05). Hypertension prevalence showed marked regional heterogeneity, from 9.5% in Kyzylorda and 9.6% in Astana to 25.3% in Akmola and 23.7% in North Kazakhstan. In adjusted models, hypertension was independently associated with older age (OR 1.894, 95% CI 1.780–2.014, *p* < 0.001), higher BMI (OR 1.597, 95% CI 1.484–1.719, *p* < 0.001), higher total cholesterol (OR 1.171, 95% CI 1.098–1.249, *p* < 0.001) and urban residence (OR 1.304, 95% CI 1.121–1.517, *p* = 0.001), while female sex was protective (OR 0.596, 95% CI 0.511–0.696, *p* < 0.001). Smoking, HbA1c, education and ethnicity were not significant after adjustment, and alcohol intake showed a statistically detectable but clinically minimal association (OR 0.997, 95% CI 0.995–0.999).

**Conclusion:**

In 2021–2022, hypertension affected roughly one in six adults in Kazakhstan and rose steeply with age. Modifiable metabolic factors, particularly excess body weight and elevated cholesterol, were the main drivers of risk, while an independent urban effect and pronounced north–south regional differences highlight the need for targeted weight and lipid management and intensified long-term risk control, especially in cities and high-prevalence northern regions.

## Background

Arterial hypertension (AH) is a leading cause of cardiovascular diseases and premature death worldwide [1]. Although frequently asymptomatic, AH is strongly associated with stroke, ischemic heart disease, heart failure, chronic kidney disease, and other non-communicable complications [2, 3]. While high-income countries have experienced modest declines in hypertension prevalence over the past two decades, many low- and middle-income countries continue to show increasing trends, largely driven by urbanization, lifestyle changes, population aging, and persistent socio-economic inequalities [4]. A wide range of sociodemographic and biological determinants influence the risk of developing hypertension, including age, gender, ethnicity, obesity, dietary habits, alcohol consumption, physical inactivity, and metabolic abnormalities [5–7].

In Kazakhstan, several studies have addressed cardiovascular health in recent years. National mortality analyses have shown persistently high cardiovascular mortality and substantial regional differences in CVD outcomes [8]. Previously, a retrospective study was conducted in Kazakhstan to assess the prevalence, morbidity, and mortality from all causes in patients with AH using large-scale nationwide medical records over a six-year period from 2014 to 2019. The results indicated an increase in prevalence, morbidity, and mortality among patients with AH [9]. However, according to this study, the true prevalence of hypertension may have been underestimated due to the retrospective nature of the research, as well as due to misclassification of cases of hypertension and incorrect coding of hypertension. In addition, recent evidence from Kazakhstan highlights substantial urban–rural disparities in cardiovascular disease incidence and prevention strategies. It should be noted that their analysis relied on administrative and surveillance data and did not include measured blood pressure, anthropometric indicators, or biochemical parameters. Consequently, the study did not evaluate the actual prevalence of arterial hypertension or its individual-level determinants [10]. These findings underscore the need for up-to-date, nationally representative epidemiological data collected using standardized and internationally comparable methods. In this context, the WHO STEPS approach provides an appropriate framework for systematically assessing hypertension-related risk factors in the population.

In this regard, this study aimed to identify sociodemographic and biological factors associated with arterial hypertension in a nationally representative sample of adults in Kazakhstan during the period from October 2021 to May 2022.

## Methods

### Data collection

In this cross-sectional study, a total of 6,720 participants aged 18 to 69 years were voluntarily recruited from all 17 regions of the country, including the cities of Almaty, Astana, and Shymkent. Recruitment took place from October 2021 to May 2022. The study used the WHO STEPS Questionnaire as a tool [11]. According to the STEPS study procedures, in Step 1, socio-demographic and behavioral information was collected. In Step 2, physical measurements, such as height, weight, and blood pressure, were obtained. In Step 3, biochemical measurements were conducted to assess blood glucose and cholesterol levels.

### Sampling design

The sample was drawn from the national sampling frame of the Bureau of National Statistics of Kazakhstan using a multistage cluster design aligned with WHO STEPS. The study targeted adults aged 18 to 69 years and stratified the population into six age groups. The required sample size was calculated with the STEPS calculator assuming 50% prevalence, 5% precision, a design effect of 1.5, and a 70% response rate, giving a minimum of 6,585 participants, while 6,720 were ultimately recruited. Selection proceeded in three stages: districts and cities were chosen as primary sampling units with probability proportional to size across all 17 regions, four primary health care facilities were randomly selected within each unit for a total of 240 facilities, and households in each facility’s catchment area were randomly sampled with one eligible adult selected per household to ensure unbiased representation by age and sex.

### Sampling weights and complex survey design

Sampling weights were constructed to reflect the multistage WHO STEPS design and to obtain nationally representative estimates. The base weight was defined as the inverse probability of selection across the three sampling stages (PSU selected by probability proportional to size, PHC facilities within PSU, and one eligible adult randomly selected per household). Weights were adjusted for non-response and post-stratified to the national age–sex distribution by region using the Bureau of National Statistics frame. PSU and stratum identifiers were retained, and all analyses incorporated weights and design variables.”

### Questionnaire

The data for the present study were collected using the WHO STEPS questionnaire, administered by an expert during an interview. The interviews were organized following participants’ prior consent and conducted by trained interviewers who received specialized training in interviewing techniques and ethical principles for data collection in medical research. Interviewers collected information on gender, age, and additional questions as listed below:

The WHO STEPS questionnaire included the following questions:

Have you ever had your blood pressure measured by a doctor or other healthcare professional?

In the past 12 months, has a doctor or other healthcare professional told you that you have high blood pressure or hypertension?

In the past two weeks, have you taken any medications for high blood pressure, prescribed by a doctor or other healthcare professional?

Have you ever had your blood sugar level measured by a doctor or other healthcare professional?

In the past 12 months, has a doctor or other healthcare professional told you that you have high blood sugar or diabetes?

In the past two weeks, have you taken any medications for diabetes, prescribed by a doctor or other healthcare professional?

Have you ever had your cholesterol level (blood lipids) measured by a doctor or other healthcare professional?

In the past 12 months, has a doctor or other healthcare professional told you that you have high cholesterol?

In the past two weeks, have you taken any medications for high cholesterol, prescribed by a doctor or other healthcare professional?

### Blood pressure measurement

The average blood pressure was measured using an OMRON HEM-7720 sphygmomanometer with appropriately sized cuffs. We calculated the average value from three consecutive blood pressure measurements. Participants were seated and allowed to rest quietly for 5 min before the measurement, in accordance with current international guidelines [12]. After this rest period, technicians conducted three measurements with an interval of 30 s to 1 min between each measurement [13].

### Laboratory measurements

Total cholesterol levels was measured, and hypercholesterolemia, defined as cholesterol levels exceeding the WHO recommended threshold of 5.0 mmol/l. Glucose levels were measured by determining the level of glycated hemoglobin, the diagnosis of diabetes was based on glycated hemoglobin was ≥ 6.5% [14].

### Definition of hypertension

Patients were considered hypertensive if they self-reported hypertension and/or if the average of the last three measurements of systolic blood pressure (SBP) during clinical examination was ≥ 140 mmHg and/or diastolic blood pressure (DBP) was ≥ 90 mmHg. According to the definition by the European Society of Cardiology (ESC) and the European Society of Hypertension (ESH), blood pressure values were classified into six categories: optimal BP, normal BP, high-normal BP, grade 1 hypertension, grade 2 hypertension, and grade 3 hypertension as defined by the ESC and ESH [15].

Participants were considered aware of their hypertension if they answered “yes” to the question, “Has a doctor or other health professional ever told you that you have high blood pressure?” Among hypertensive participants who were aware of their condition, a person was considered treated for hypertension if they reported taking medications to lower their blood pressure. Among those reporting antihypertensive therapy, hypertension was considered controlled when SBP was < 140 mmHg and DBP was < 90 mmHg [16]. Participants were classified as having diabetes if they had HbA1c levels ≥ 6.5%.

### Covariates

Body mass index (BMI) was calculated as weight in kilograms divided by the square of height in meters. Overweight was defined as a BMI of ≥ 25 and < 30 kg/m², and obesity was defined as a BMI of ≥ 30 kg/m² [13]. Tobacco and alcohol consumption, educational level, employment status, etc., were collected using questionnaires.

### Statistical analysis

Data were analyzed using IBM SPSS Statistics version 22 (IBM Corp., USA). All data were checked for missing values and anomalies. Missing data were assessed prior to analysis. Observations with incomplete data for key variables were excluded using listwise deletion. The proportion of missing data was low and was therefore unlikely to introduce bias. Descriptive statistics were used to characterize the sample: means, standard deviations, medians, interquartile ranges, as well as proportions and frequencies for categorical variables, were calculated. The normality of continuous variables was assessed using the Shapiro–Wilk test, and most continuous variables demonstrated a normal distribution; therefore, the independent samples t-test was used for group comparisons (urban vs. rural participants, men vs. women). For categorical data analysis, the chi-square test was applied. The relationship between continuous variables was evaluated using Pearson’s correlation coefficient. Logistic regression was used to assess risk factors for hypertension, including variables such as age, body mass index (BMI), cholesterol level, and place of residence.Covariates were selected a priori based on established hypertension determinants and included age, sex, BMI, total cholesterol, HbA1c, smoking status, alcohol consumption, education, ethnicity, and place of residence. Multicollinearity was assessed using variance inflation factors; no relevant collinearity was detected. Interaction terms (sex×residence and age×residence) were tested and were not statistically significant, therefore they were not retained in the final model. Regression results are presented as ORs with 95% CIs accounting for survey design. All statistical tests were considered significant at a p-value < 0.05.

## Results

### Descriptive characteristics of the study population

A total of 6,720 adults aged 18 to 69 years were included in the analysis. Urban residents accounted for 4,401 participants (65.5%) and rural residents for 2,319 (34.5%). Urban areas had a higher proportion of men than rural areas (54.3 vs. 42.0%), while women were more prevalent in rural settings (58.0 vs. 45.7%), *p* < 0.001 (Table 1). The urban sample was younger overall, whereas rural areas included relatively more adults aged 55 years and older, *p* < 0.001.

**Table 1:** General characteristics of study participants

Characteristic	Urban (*n* = 4401, 65.5%)	Rural (*n* = 2319, 34.5%)	*p*-value
**Gender**			< 0.001
Male	2391 (54.3%)	974 (42%)	
Female	2010 (45.7%)	1345 (58%)	
**Age**			< 0.001
18–24 years	687 (15.6%)	232 (10%)	
25–34 years	1166 (26.5%)	475 (20.5%)	
35–44 years	1026 (23.3%)	500 (21.6%)	
45–54 years	791 (18%)	477 (20.6%)	
55–64 years	549 (12.5%)	472 (20.4%)	
65 years and older	182 (4.1%)	163 (7%)	
**Smoking Status**			
Yes	941 (21.4%)	343 (14.8%)	
No	3460 (78.6%)	1976 (85.2%)	
**Alcohol Consumption Frequency (last 12 months)**			< 0.001
Daily	13 (0.3%)	7 (0.3%)	
5–6 times per week	9 (0.2%)	4 (0.2%)	
3–4 times per week	36 (0.8%)	11 (0.5%)	
1–2 times per week	284 (6.5%)	74 (3.2%)	
1–3 times per month	521 (11.8%)	151 (6.5%)	
Less than once per month	1243 (28.2%)	509 (21.9%)	
No response	2295 (52.1%)	1563 (67.4%)	
**Ethnicity**			< 0.001
Kazakh	2886 (65.6%)	1488 (64.2%)	
Russian	1028 (23.4%)	522 (22.5%)	
Uzbek	95 (2.2%)	107 (4.6%)	
Ukrainian	79 (1.8%)	31 (1.3%)	
Uyghur	36 (0.8%)	1 (0%)	
Tatar	67 (1.5%)	50 (2.2%)	
Other	205 (4.7%)	118 (5.1%)	
Refused to answer	5 (0.1%)	2 (0.1%)	
**Education Level**			< 0.001
No formal schooling	46 (1%)	26 (1.1%)	
Completed primary education (4 years)	9 (0.2%)	2 (0.1%)	
Completed secondary education (9 years)	245 (5.6%)	171 (7.4%)	
Completed secondary education (11 years)	1115 (25.3%)	676 (29.2%)	
Higher education	2112 (48%)	961 (41.4%)	
Graduate education (Master/PhD)	797 (18.1%)	447 (19.3%)	
Refused to answer	77 (1.7%)	36 (1.6%)	
**BMI**			< 0.05
< 18.5 Underweight	150 (3.4%)	61 (2.6%)	
18.5–25 Normal	1795 (40.8%)	785 (33.9%)	
25–30 Overweight	1583 (36%)	905 (39%)	
30–35 Obesity Class 1	661 (15%)	391 (16.9%)	
35–40 Obesity Class 2	160 (3.6%)	141 (6.1%)	
> 40 Obesity Class 3	52 (1.2%)	36 (1.6%)	
**Hip Circumference (cm)**	101 ± 14	100 ± 14	< 0.05
**Waist Circumference (cm)**	87 ± 15	88 ± 15	< 0.05
**Systolic BP (mmHg)**	122 ± 18	124 ± 20	< 0.05
**Diastolic BP (mmHg)**	80 ± 12	82 ± 16	< 0.05
**Cholesterol (mmol/L)**	4.9 ± 1.1	5 ± 1.2	< 0.05
**Glycated Hemoglobin (%)**	5.5 ± 0.9	5.7 ± 1.2	< 0.05
**Vegetable Consumption per Week**	5.1 ± 2.0	4.8 ± 2.1	< 0.05
**Fruit Consumption per Week**	3.9 ± 2.6	4.0 ± 2.2	0.054

Behavioral patterns differed by residence. Current smoking was more common in urban than rural residents (21.4 vs. 14.8%, *p* < 0.001). Alcohol use at least 1 to 2 days per week was also reported more often in urban settings (6.5 vs. 3.2%, *p* < 0.01), although non-response for alcohol intake was substantial and is addressed in the limitations.

Metabolic and clinical profiles were slightly less favorable in rural residents. Rural participants had higher mean BMI and a higher prevalence of obesity class 2 (6.1 vs. 3.6%). They also showed modestly higher mean systolic and diastolic blood pressure, total cholesterol, and HbA1c levels (*p* < 0.05).

### National prevalence of hypertension

In the national sample, the overall crude prevalence of hypertension was 16.0% (1,072 of 6,720, 95% CI 15.1 to 16.8). Hypertension prevalence increased sharply with age, from 3.0% in adults aged 18 to 24 years to 46.7% among adults aged 65 years and older (Table 2). Age-related shifts were also evident across blood pressure categories. Optimal blood pressure was most frequent in the youngest group (61.5%) and declined steadily with age, while stage 1 to 3 hypertension rose from 2.8% in those aged 18 to 24 years to 46.6% in those aged 65 years and older.

**Table 2:** Comparative blood pressure indicators by gender and age categories

Indicator	Gender		Age category					
	Male	Female	18–24	25–34	35–44	45–54	55–64	64 and older
**Systolic BP**	126 ± 17	119 ± 19	114 ± 13	116 ± 14	120 ± 16	126 ± 19	135 ± 21	139 ± 21
**Diastolic BP**	82 ± 12	79 ± 15	76 ± 11	77 ± 10	80 ± 11	84 ± 19	86 ± 12	85 ± 12
**Pulse**	43,2 ± 12,6	40,0 ± 15,9	38,1 ± 11,1	38,9 ± 10,7	39,3 ± 10,7	41,6 ± 19,4	48,2 ± 15,3	53,8 ± 15,8
**Blood Pressure Category n (%)**								
Optimal (≤ 120/80 mmHg)	1049 (31,2)	1776 (52,9)	565 (61,5)	865 (52,7)	712 (46,7)	432 (34,1)	198 (19,4)	53 (15,4)
Normal (120–129/80–84 mmHg)	1180 (35,1)	757 (22,6)	270 (29,4)	514 (31,3)	480 (31,5)	377 (29,7)	240 (23,5)	56 (16,2)
High-Normal (130–139/85–89 mmHg)	528 (15,7)	358 (10,7)	56 (6,1)	178 (10,8)	169 (11,1)	209 (16,5)	199 (19,5)	75 (21,7)
Hypertension Stage 1 (140–159/90–99 mmHg)	452 (13,4)	322 (9,6)	26 (2,8)	71 (4,3)	137 (9)	179 (14,1)	255 (25)	106 (30,7)
Hypertension Stage 2 (160–179/100–109 mmHg)	120 (3,6)	100 (3)	2 (0,2)	11 (0,7)	20 (1,3)	50 (3,9)	97 (9,5)	40(11,6)
Hypertension Stage 3 (≥ 180/≥110 mmHg)	36 (1,1)	42 (1,3)	0	2 (0,1)	8 (0,5)	21 (1,7)	32 (3,1)	15 (4,3)

### Urban–rural and sex differences in blood pressure distribution

Men had higher average systolic and diastolic blood pressure than women (126 ± 17 vs. 119 ± 19 mmHg for systolic, and 82 ± 12 vs. 79 ± 15 mmHg for diastolic). Consistent with these mean differences, optimal blood pressure was more frequent in women than men (52.9 vs. 31.2%), whereas high-normal blood pressure and stage 1 hypertension were more prevalent in men (Table 2). Despite the younger age structure in urban settings, hypertension was not lower in cities. Urban residents more frequently reported smoking and alcohol use, while rural residents showed higher BMI and slightly higher measured blood pressure. These cross-residence contrasts motivated multivariable modeling to determine which factors were independently associated with hypertension.

### Regional variation in hypertension prevalence

**Fig. 1:**
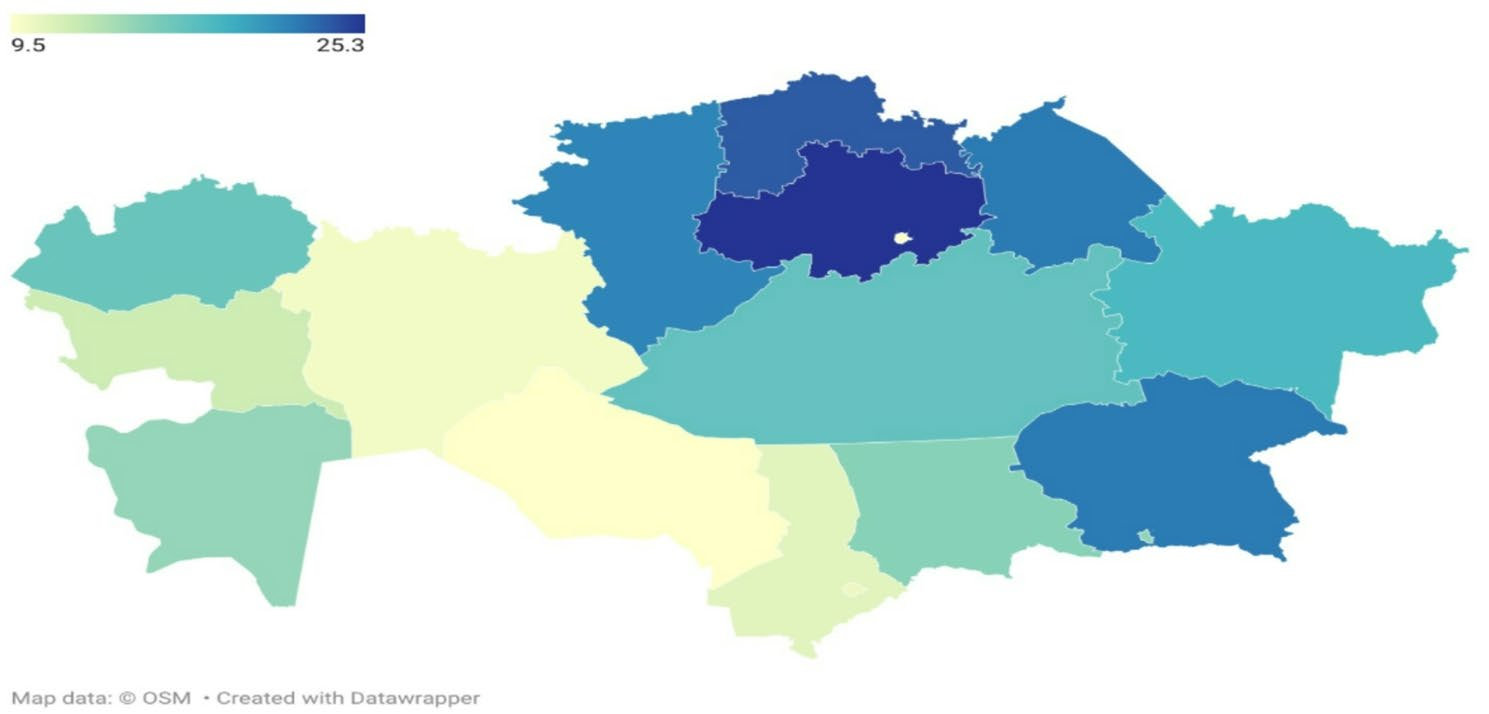
Prevalence of Hypertension in Different Regions of Kazakhstan

The lowest prevalence was observed in Kyzylorda (9.5%) and Astana (9.6%), while the highest prevalence was recorded in Akmola (25.3%) and North Kazakhstan (23.7%). Almaty city showed a prevalence of 15.0%, higher than Astana. Overall, lower rates tended to cluster in southern and western regions such as Kyzylorda and Mangystau, whereas higher rates were found in northern regions including Akmola and North Kazakhstan (Fig. 1).

### Awareness and treatment indicators from WHO STEPS

Key STEPS indicators of screening, awareness, and treatment were broadly similar between urban and rural participants. Most respondents reported ever having their blood pressure measured (91.9% in urban and 92.3% in rural areas). Around three fifths recalled being told of high blood pressure within the past 12 months, and roughly half of those aware reported taking antihypertensive medications in the prior two weeks, with no statistically meaningful urban–rural differences (Table 3). Patterns were comparable for glucose and cholesterol screening and treatment indicators.

**Table 3:** Comparative characteristics of participants based on WHO STEPS survey

Characteristic	Urban (*n*, %)	Rural (*n*, %)	*p*-value
**Age**			≤ 0.05
18–24 years	21 (3.5)	7 (1.5)	
25–34 years	59 (9.7)	25 (5.4)	
35–44 years	106 (17.5)	59 (12.7)	
45–54 years	144 (23.8)	106 (22.7)	
55–64 years	204 (33.7)	180 (38.6)	
64 years and older	72 (11.9)	89 (19.1)	
Have you ever had your blood pressure measured by a doctor?			0.234
Yes	557 (91.9)	430 (92.3)	
No	49 (8.1)	36 (7.7)	
In the last 12 months, has a doctor told you that you have high blood pressure?			0.074
Yes	353 (58.3)	280 (60.1)	
No	253 (41.7)	186 (39.9)	
Have you taken any medication for high blood pressure in the last two weeks?			0.391
Yes	280 (46.2)	222 (47.6)	
No	326 (53.8)	244 (52.4)	
Have you ever had your blood sugar level measured by a doctor?			0.121
Yes	433 (71.5)	346 (74.2)	
No	173 (28.5)	120 (25.8)	
Are you currently taking insulin for diabetes treatment?			0.119
Yes	15 (2.5)	7 (1.5)	
No	591 (97.5)	459 (98.5)	
In the last 12 months, has a doctor or other health worker told you that you have high blood sugar?			0.098
Yes	89 (14.7)	62 (13.3)	
No	517 (85.3)	404 (86.7)	
Have you ever had your cholesterol measured by a doctor or other health worker?			0.087
Yes	380 (62.7)	306 (65.7)	
No	226 (37.3)	160 (34.3)	
In the last two weeks, have you taken medication for high cholesterol?			0.431
Yes	40 (6.6)	32 (6.9)	
No	566 (93.4)	434 (93.1)	
In the last 12 months, has a doctor told you that you have high cholesterol?			0.592
Yes	92 (15.2)	92 (19.7)	
No	514 (84.8)	374 (80.3)	

### Multivariable analysis of hypertension risk factors

In the fully adjusted logistic regression model (Table 4), age and adiposity were the dominant correlates of hypertension. Each modeled increment in age was associated with almost two-fold higher odds of hypertension (OR 1.894, 95% CI 1.780 to 2.014, *p* < 0.001). Higher BMI was linked to substantially higher odds (OR 1.597, 95% CI 1.484 to 1.719, *p* < 0.001). Total cholesterol showed an independent positive association with hypertension (OR 1.171, 95% CI 1.098 to 1.249, *p* < 0.001). Urban residence remained an independent predictor after accounting for metabolic and sociodemographic factors (OR 1.304, 95% CI 1.121 to 1.517, *p* = 0.001), indicating that hypertension risk in cities is not explained solely by BMI, cholesterol, or age distribution.

**Table 4:** Logistic regression results for assessing hypertension risk factors

Variable	Coef. (B)	Standard error (SE)	Wald (W)	*p*-value	OR	95% Confidence interval
Urban vs. Rural	0.266	0.077	11.871	0.001	1.304	1.121–1.517
BMI	0.468	0.038	155.287	< 0.001	1.597	1.484–1.719
Gender	-0.517	0.079	42.812	< 0.001	0.596	0.511–0.696
Education	-0.001	0.004	0.074	0.786	0.999	0.992–1.006
Ethnicity	0.003	0.011	0.089	0.765	1.003	0.981–1.026
Smoking	-0.142	0.097	2.129	0.145	0.867	0.717–1.050
Alcohol (last 12 months)	-0.003	0.001	7.787	0.005	0.997	0.995–0.999
Age	0.638	0.032	409.878	< 0.001	1.894	1.780–2.014
Total Cholesterol	0.158	0.033	23.133	< 0.001	1.171	1.098–1.249
Glycated Hemoglobin	0.022	0.033	0.434	0.510	1.022	0.958–1.089

Sex showed an inverse association (OR 0.596, 95% CI 0.511 to 0.696, *p* < 0.001), consistent with the higher mean blood pressure and less favorable category distribution in men. Smoking, ethnicity, education, and HbA1c were not independently associated with hypertension after adjustment. Alcohol consumption showed a statistically significant but clinically weak association (OR 0.997, 95% CI 0.995 to 0.999). Model diagnostics indicated no meaningful multicollinearity, and interaction terms tested for age by residence and sex by residence were not significant.

## Discussion

This national WHO STEPS survey provides an updated picture of hypertension risk in Kazakhstan based on standardized measurements and a representative sample. The analysis shows that hypertension in adults aged 18 to 69 years is primarily associated with older age, higher BMI, elevated total cholesterol, and urban residence. At the same time, substantial regional variability was observed, with higher prevalence in northern regions and lower prevalence in southern and western areas.

Age emerged as the strongest non-modifiable correlate, with a near twofold increase in odds across the modeled age increment. This aligns with international and regional evidence that blood pressure rises steadily with aging and that older adults carry the highest burden of hypertension complications [17, 18]. The steep age gradient in our data also mirrors patterns reported in other STEPS-based surveys and global analyses [3, 19]. These findings support prioritizing early detection and longitudinal follow-up in older age groups.

Among modifiable drivers, excess body weight had a major independent role. The high odds associated with BMI are consistent with established pathophysiological links between adiposity, insulin resistance, sympathetic activation, and renal sodium retention [20, 21]. Evidence from intervention studies indicates that sustained weight reduction leads to meaningful decreases in hypertension risk and improves blood pressure control [22]. In Kazakhstan, where overweight and obesity are common, this reinforces the centrality of weight management in prevention strategies.

Total cholesterol was also independently associated with hypertension. This finding fits the broader cardiometabolic clustering described in many populations, where dyslipidaemia coexists with obesity and elevated blood pressure 29, 31. From a clinical perspective, it argues for integrated risk-factor management rather than treating hypertension in isolation.

An important result is the persistence of the urban residence effect after adjusting for age, BMI, lipids, and other covariates. Previous multicountry research, including the PURE study, has shown that urban settings in middle-income countries may have higher hypertension awareness and treatment but also higher exposure to lifestyle and environmental risks [16, 23]. In Kazakhstan, administrative data suggested urban living as a risk context, but lacked measured biological determinants [9, 10]. Our measured STEPS data confirm that the urban effect is not solely explained by metabolic profile or age distribution, pointing to additional urban-related exposures that need direct assessment in future studies.

Smoking and alcohol use were more prevalent in urban participants in descriptive analyses, yet neither showed a meaningful independent association with hypertension after multivariable adjustment. This pattern is plausible. First, smoking and alcohol are strongly age- and sex-patterned behaviors and may lose independent predictive value once major cardiometabolic determinants are included. Second, the association between tobacco or alcohol and hypertension is inconsistent across settings, and large reviews highlight heterogeneity and residual confounding [19]. Third, in our dataset al.cohol intake had substantial non-response, which likely attenuated detectable effects in regression and argues for cautious interpretation. We therefore view these behaviors as important public-health targets, but not the dominant independent predictors in this specific model. Similar reasoning applies to HbA1c. While dysglycaemia is linked to vascular risk, its contribution may be mediated through BMI and age, leaving no independent effect once these pathways are accounted for [19, 24].

Regional variation was pronounced. Higher prevalence in Akmola and North Kazakhstan and lower prevalence in Kyzylorda and Astana indicate a clear north–south gradient. We refrain from attributing these differences to specific causes because socioeconomic structure, dietary sodium patterns, climate, and healthcare access were not directly measured at the regional level in this survey. Still, the observed gradient is compatible with prior national CVD mortality analyses showing regional heterogeneity in cardiovascular outcomes [8], and with evidence from other STEPS settings where disparities in access and preventive care contribute to uneven hypertension burden [25]. Future work linking STEPS outcomes to regional socioeconomic and health-system indicators would be valuable to test these hypotheses.

From a policy and practice standpoint, the results point to a straightforward priority set. Metabolic risk reduction should be the core prevention axis, including population measures to reduce obesity, improve diet quality, and strengthen lipid control [20, 22]. Urban areas and high-prevalence northern regions warrant intensified screening and structured follow-up given the independent urban effect and the regional peaks. STEPS indicators suggest that screening uptake is high and broadly similar across urban and rural settings. The main opportunity likely lies in improving sustained treatment adherence and long-term control, which is a common challenge internationally [26]. Integrating hypertension control with stroke-prevention messaging and risk management is also essential, given the strong causal link between uncontrolled hypertension and stroke [27–29].

Overall, this STEPS-based assessment clarifies that hypertension in Kazakhstan is driven mainly by age-related risk and modifiable metabolic factors, with additional urban and regional heterogeneity. Targeted, region-tailored programs that combine early detection with strong weight and lipid management are likely to yield the greatest population benefit.

## Conclusion

In conclusion, during the 2021–2022 study period, hypertension in Kazakhstan was primarily driven by older age, higher BMI, and elevated total cholesterol levels, with urban residence also contributing to increased risk. Although behavioral factors such as smoking and alcohol use were more common among urban participants, they did not remain significant predictors after adjustment. Marked regional differences and the less favorable cardiometabolic profile observed in rural populations underscore substantial health disparities. These findings highlight the need for region-specific and population-tailored strategies for hypertension prevention and management, with particular emphasis on metabolic risk reduction and improved access to healthcare services.

### Study limitations

Our study has certain limitations. We presented our results using a cross-sectional design, which makes it challenging to assess complications related to hypertension over long-term follow-up, which can be evaluated in future studies. Although analyses accounted for complex sampling, some behavioral variables had non-response that may have introduced residual uncertainty; however, sensitivity analyses suggested no material impact on the main conclusions.

## Data Availability

All available data was indicated within manuscript text.

## Abbreviations

AH: Arterial Hypertension
BMI: Body Mass Index
BP: Blood Pressure
DBP: Diastolic Blood Pressure
ESC: European Society of Cardiology
ESH: European Society of Hypertension
HbA1c: Glycated Hemoglobin
AH: Hypertension
OR: Odds Ratio
SBP: Systolic Blood Pressure
SD: Standard Deviation
STEPS: Stepwise Approach to Chronic Disease Risk Factor Surveillance (WHO)
WHO: World Health Organization
